# Nutraceuticals and Their Potential to Treat Duchenne Muscular Dystrophy: Separating the Credible from the Conjecture

**DOI:** 10.3390/nu8110713

**Published:** 2016-11-09

**Authors:** Keryn G. Woodman, Chantal A. Coles, Shireen R. Lamandé, Jason D. White

**Affiliations:** 1 Murdoch Childrens Research Institute, Royal Children’s Hospital, Parkville 3052, Australia; kerynwoodman@icloud.com (K.G.W.); chantal.coles@mcri.edu.au (C.A.C.); shireen.lamande@mcri.edu.au (S.R.L.); 2 Faculty of Veterinary and Agricultural Science, The University of Melbourne, Parkville 3010, Australia; 3 Department of Pediatrics, The University of Melbourne, Parkville 3010, Australia

**Keywords:** Duchenne muscular dystrophy, Becker muscular dystrophy, muscle, nutraceuticals, *mdx*

## Abstract

In recent years, complementary and alternative medicine has become increasingly popular. This trend has not escaped the Duchenne Muscular Dystrophy community with one study showing that 80% of caregivers have provided their Duchenne patients with complementary and alternative medicine in conjunction with their traditional treatments. These statistics are concerning given that many supplements are taken based on purely “anecdotal” evidence. Many nutraceuticals are thought to have anti-inflammatory or anti-oxidant effects. Given that dystrophic pathology is exacerbated by inflammation and oxidative stress these nutraceuticals could have some therapeutic benefit for Duchenne Muscular Dystrophy (DMD). This review gathers and evaluates the peer-reviewed scientific studies that have used nutraceuticals in clinical or pre-clinical trials for DMD and thus separates the credible from the conjecture.

## 1. Introduction

Duchenne Muscular Dystrophy (DMD) is a fatal X-linked muscle disease affecting 1 in 3500 boys (comprehensively reviewed in [1,2,3]). DMD is caused by mutations (predominantly deletions) in the dystrophin gene (*DMD*, locus Xp21.2) [4] that result in the absence or severe reduction of the cytoskeletal protein dystrophin [5]. The much milder Becker Muscular Dystrophy (BMD) is typically the result of in frame deletions in the same gene. In DMD, the entire dystrophin glycoprotein complex (DGC), which links the actin cytoskeleton to the extracellular matrix, is lost, and muscle is susceptible to damage caused by repeated muscle contractions. This continuous damage causes progressive muscle degeneration; resident satellite cells are activated in a continuous cycle of muscle damage and repair, ultimately depleting the satellite cell population critical for muscle repair [6]. Clinically, patients typically lose ambulation by their teens and if the disease is left untreated, succumb to cardiac or respiratory failure with the mean age of death at 19 years [7]. However, with current interventions including corticosteroid therapy, and respiratory, cardiac, orthopedic and rehabilitative care, survival can be prolonged to the third and even fourth decade of life [7,8,9,10,11,12,13,14,15,16,17].

Corticosteroids slow the decline in muscle strength and function [8,9,18,19,20,21,22,23,24,25] and are used to prolong ambulation and stabilize pulmonary function [20,21,25]. Adverse side effects are associated with corticosteroid therapy in DMD patients and can include weight gain [26], growth retardation [27,28], bone demineralization [22,29,30,31] (and therefore high risk of fractures), hypertension [29], behavioral issues [8,29,32] and delayed onset of puberty [33]. Therefore, the type of corticosteroid prescribed, along with the dosage and treatment regime varies between patients depending on their tolerance to the medication (see [2,3] for reviews).

Potential therapeutics that are currently in development for treating DMD include exon skipping to restore the codon reading frame and produce partially functional truncated dystrophin protein, gene therapy and cell transplantation strategies to replace the mutant *DMD* gene. Initial cell transplantation strategies centered on primary myoblasts or satellite cells but more recent research has highlighted the contribution of other cell types to regeneration in skeletal muscle has led to the consideration of other atypical stem cells [34,35]. The greatest potential seems to be with mesangioblasts [36,37], pericytes [38,39] and CD133+ cells [40,41]. More recently induced pluripotent stem cells (iPSCs) are also attracting much attention with the optimization of conditions for conversion to skeletal muscle precursors [42,43]. Another approach aimed at compensating for the loss of dystrophin is the use of small molecules to induce stop codon read through, or upregulate the dystrophin homolog utrophin (for excellent reviews of these technologies, see [44,45]). These therapies are promising and many have reached clinical trials [46,47,48,49]; however, the results have been disappointing in some cases and variable in others [50,51], and it is clear that these approaches will need extensive optimization before they are available for routine clinical use. There is an urgent need for novel treatment options for DMD patients; however, in the interim, nutraceuticals could potentially be used to alleviate inflammation and oxidative stress which contribute to disease pathology.

### Could Nutraceuticals Fill the Current Void in Treatment Options for DMD Patients?

There is no US Food and Drug Administration (FDA) approved definition of a nutraceutical; however, the Canadian definition is “a compound within a food that can be isolated and purified and sold that has the potential to benefit health and treat chronic disease” [52]. A 2007 report by the National Institutes of Health showed that $33.9 billion dollars were spent per year in the US alone on Complementary and Alternative Medicine (CAM), including nutraceutical products [53]. In Australia, a 2007 report revealed that the annual out of pocket figure for CAM nationwide is AU $4.13 billion dollars [54]. With a growing trend to seek out alternative therapies, it is not surprising that parents of children with devastating incurable neuromuscular disorders such as DMD are looking towards alternative therapies and nutraceuticals in the hope that they will improve their child’s condition. In Canada 20% of Duchenne caregivers report administering CAM to their DMD child in conjunction with traditional medicine [55], and in the US 80% of surveyed DMD and Becker muscular dystrophy caregivers had given CAM to their patients in conjunction with their traditional treatment [56]. Whilst some caregivers believe that nutraceuticals have improved the condition of their DMD patient/child, much of this “evidence” is purely anecdotal. Therefore, the aim of this review is to critically evaluate peer-reviewed scientific data on nutraceutical therapies for DMD.

## 2. DMD Pathogenesis and the *mdx* Mouse Model

DMD pathogenesis is complex and has been reviewed extensively [57]. The major pathogenic pathways targeted by nutraceutical therapies are inflammation and oxidative stress. Dystrophin loss results in constant bouts of muscle fiber damage and necrosis followed by regeneration. The muscle damage triggers an influx of inflammatory cells which clear necrotic tissue, and release pro-inflammatory cytokines that recruit more immune cells and further exacerbate the pathology [58,59,60]. The disruption to muscle homeostasis also triggers oxidative stress mechanisms which contribute to the phenotype [61,62,63]. Many nutraceuticals have anti-inflammatory or antioxidant properties which could lessen pathology and provide patients with some functional improvements.

Whilst some studies have assessed nutraceutical therapies in DMD patients, most published research uses the *mdx* mouse model of DMD [64]. Briefly, the *mdx* mutation is a premature termination codon in exon 23 of the *Dmd* gene, resulting in the absence/severe deficiency of dystrophin protein [65,66]. *Mdx* mice exhibit an acute onset of pathology at approximately three weeks of age characterized by elevated serum levels of creatine kinase and pyruvate kinase [66,67], and muscle necrosis and regeneration similar to that observed in DMD patients [67,68]. After eight weeks of age, the pathology subsides to a chronic level which is maintained throughout the lifespan of the *mdx* mouse. This chronic level of disease pathology in *mdx* mouse muscle is much less severe than that observed in human DMD patients, with the exception of the *diaphragm* muscle [69]. For more comprehensive reviews of the *mdx* mouse see [57,70,71].

## 3. Targeting Oxidative Stress

Oxidative stress has been linked to numerous diseases and it is also an important contributor to DMD pathology [61,62,72,73,74,75]. Markers of oxidative stress including by-products of lipid peroxidation and protein oxidation are elevated in DMD patients [61,76] and in *mdx* mice [62,77] and isolated dystrophin deficient myotubes from *mdx* mice are more susceptible to oxidative damage [78].

Oxidative stress results from an imbalance in the production of reactive oxygen species (ROS) and their removal by specific defense systems, namely antioxidants. Unless the ROS are removed by antioxidants, ROS accumulation occurs which ultimately leads to cell death and tissue degeneration. The sources of oxidative stress in DMD are thought to include inflammatory cells, NAD(P)H oxidase, altered mitochondrial function or directly from ROS producing enzymes (inducible nitric oxide synthase, iNOS), and insufficient cell stress responses [79]. The antioxidant defense system is comprised of antioxidant enzymes including Cu,Zn-superoxide dismutase (SOD1), Mn-superoxide dismutase (SOD2), glutathione peroxidase, and catalase (reviewed in [63]). These antioxidant enzymes catalyze reactions that convert ROS to less reactive species thus protecting the system from oxidative damage. Antioxidants can act either directly by scavenging free radicals, or indirectly by increasing exogenous cellular defenses including activation of the nuclear factor erythroid derived 2-related factor 2 (Nrf2) transcription factor pathway. Nrf2 is important in protecting cells from oxidative stress and inflammation [80]. Whilst antioxidants are important for clearing ROS, a homeostatic balance is required between ROS and the antioxidants; high levels of the antioxidant SOD1 in mice lead to a muscular dystrophy phenotype [81]. Neuronal nitric oxide synthase (nNOS) is a component of the DGC and nNOS levels are dramatically reduced in DMD [82]. As a consequence production of the anti-inflammatory molecule nitric oxide (NO) is also severely reduced. Transgenic expression of nNOS in the *mdx* mouse normalizes NO production, and reduces muscle membrane damage and inflammation [83].

As oxidative stress exacerbates DMD pathology, nutraceuticals with antioxidant capabilities could be beneficial in DMD. Some antioxidant nutraceuticals trialed in DMD include Coenzyme Q10, melatonin and preparations of traditional Chinese medicine.

### 3.1. Coenzyme Q10

Coenzyme Q10 (CoQ10), or ubiquinone has many roles central to metabolic function. CoQ10 is located in the inner membrane of the mitochondria where its main function is to accept electrons for the nicotinamide adenine dinucleotide dehydrogenase (NADH) and succinate dehydrogenase (SDH) complexes of the respiratory chain [84]. When CoQ10 is exogenously administered into mitochondria, it can increase the oxidative capacity of NADH and assists in metabolically supporting muscle [85]. In addition to its role in the respiratory chain, CoQ10 is a powerful antioxidant that can reduce ROS accumulation in muscle and modulate the mitochondrial transition pore to prevent calcium accumulation in muscle [84].

Initial clinical trials of CoQ10 administered 100 mg CoQ10 daily for three months to 15 patients with various neuromuscular disorders [86]. One DMD and two BMD patients self-reported physical improvements. Blood CoQ10 levels were not significantly increased compared to the placebo group in all patients leading the authors to conclude that the 100 mg dosage was too low. A second study [87] with 12 ambulant DMD patients aged 5–10 years (who had been taking prednisone for at least six months prior to the trial) used an initial dose of 400 mg with a subsequent daily 100 mg until participants reached a CoQ10 plasma level of 2.5 ug/mL. There was no placebo group in this open label study. Once participants reached this minimum CoQ10 plasma level they continued on that dose for the six-month trial period. Physiological measures were assessed to determine if the CoQ10 treatment improved Quantitative Muscle Testing (QMT) scores (including measurements of grip, muscle extension and flexion described in [88]). Functional tests were also analyzed, including time to climb steps and time to run/walk 10 m. Of the 12 participants, nine showed an increase in QMT scores of between 2% and 10%. Interestingly the patients that did not show an improvement were aged between 7.5 and 8.4 years old. Patients with DMD generally succumb to cardiac failure and therefore this study also measured the efficacy of CoQ10 in cardiac muscle. Cardiac measures were recorded including; ejection fraction, left ventricular internal diameter and posterior wall thickness by electrocardiogram during the CoQ10 trial; however, no significant improvements were observed. The conclusions from this small scale study were positive, yet due to small numbers (12 patients) and the short duration of treatment, a larger trial is warranted. This larger Cooperative International Neuromuscular Research Group (CINRG) trial is currently in the recruitment phase [89].

CoQ10 was generally well tolerated in the published trials and the only adverse effect noted was a headache of moderate intensity due to high plasma CoQ10 levels (7.37 ug/mL) in one patient. This adverse effect was resolved by decreasing the dose [87]. Toxicity assessment in a double-blinded trial for patients on three different doses of CoQ10 indicate that healthy adults can safely take up to 900 mg CoQ10 daily for four weeks without adverse side-effects [90].

Whilst it is unlikely that CoQ10 will replace the current corticosteroids treatment for DMD patients, it could be a valuable addition to help preserve muscle strength and function in patients who have adverse side effects from corticosteroids. More conclusive data on the efficacy of CoQ10 in DMD should come from the larger CINRG trial in progress.

### 3.2. Melatonin

Melatonin (*N*-acetyl-5-methoxytryptamine) is a hormone produced in plants and in the pineal gland of mammals [91]. Melatonin plays vital roles in multiple homeostatic processes including regulation of circadian rhythm, seasonal reproductive regulation, stimulation of the immune system and regulation of blood pressure (reviewed in [92]). Melatonin was described as an antioxidant in 1993 [93] and since then it has been shown to reduce free-radical production within the mitochondria, stimulate antioxidant enzymes, promote glutathione synthesis (another antioxidant) and inhibit enzymes such as NOS that produce free-radicals which cause oxidative damage (reviewed in [94]). Collectively these actions, and the fact that melatonin is lipophilic and easily passes through cell membranes and the blood-brain barrier, make it a potent antioxidant. In skeletal muscle, melatonin preserves mitochondrial function [95,96] and regulates calcium homeostasis during muscle contraction [97,98].

A pre-clinical study treated *mdx^5Cv^* mice with either daily intra-peritoneal injections of melatonin (30 mg/kg bodyweight), one melatonin subcutaneous implant (18 mg) or three implants (54 mg) for 12 days [99]. Mice on the higher implant dose and the daily injection group had reduced serum creatine kinase (CK) [99]. The *triceps* muscle contracted and relaxed faster in the daily melatonin injected mice compared to controls. Glutathione was elevated in all melatonin groups, with the ratio between oxidative-to-reduced glutathione decreased in the high dosage and the daily treatment. This ratio indicates a healthier redox status and decreased oxidative stress in muscle.

In a clinical trial 10 DMD patients aged 12.8 ± 0.98 years who had been treated with prednisone for at least five years were administered melatonin (a 60 mg dose at 9:00 p.m. and a 10 mg dose at 9:00 a.m.) and outcomes were measured at three, six and nine months [100,101]. After three months, the oxidative-to-reduced glutathione ratio was significantly reduced compared to healthy-matched controls, and the reduced ratios were maintained for the remaining six months of treatment. SOD levels were reduced to control levels after three months of treatment and these levels were maintained out to nine months. Serum CK levels were reduced in the melatonin-treated patients indicating there was less muscle damage. Importantly, the melatonin treatment reduced markers of oxidative stress and pro-inflammatory cytokines including Il-1β, IL-2, IL-6, TNF-α and INF-γ. Inflammation and anti-inflammatory compounds are discussed in detail later in this review.

Whilst these trials are promising and demonstrate melatonin’s potent antioxidant effects, there are few data in the clinical trials related to pathology and muscle function. The only parameter that indicates less muscle damage is the reduced serum creatine kinase [101]. Muscle biopsies were not analyzed to determine if there was reduced pathology, and no quantitative muscle assessments were used to assess if there were physical improvements in muscle function or performance. To conclusively determine the treatment potential of melatonin in DMD future trials need to assess the impact of melatonin treatment on disease pathology and functional muscle parameters. Melatonin has an excellent safety profile in adults with both short and long term use and has only been associated with mild side effects such as such as dizziness, headache, nausea and sleepiness [102]. It is important to note that there are no long-term studies assessing melatonin safety in children and adolescents and as such, until further studies in DMD determine whether it is therapeutically useful, supplementation is not advised.

### 3.3. Traditional Chinese Medicine

Traditional Chinese Medicine is becoming an increasingly popular alternative therapy and there has been anecdotal evidence suggesting that it can be used to slow disease progression in DMD patients. To determine if the anecdotal evidence could be substantiated, a pilot study assessed a group of 10 DMD patients that had undergone some form of traditional Chinese medicine which included either herbs, acupuncture or a combination of the two [103]. This small scale study was very limited, and did not provide information on the brands of herbs used, their purification or their dosages. There were no functional assessments and only brief clinical observations were made. No definitive conclusions could be made from this study. Following this report, the herbs (of unknown source and dosages) were obtained and analyzed [104]. This study demonstrated that the Chinese herbal extracts used in the initial trial possessed glucocorticoid activity, explaining why they could have shown beneficial effects in DMD patients.

The only other study to assess the use of Chinese herbal medicine treated *mdx* mice with an over the counter supplement, Prostandim (from LifeVantage Corp, San Diego, CA, USA), which contained *Bacopa monniera* extract, silymarin, Indian ginseng, green tea extract and curcumin [105]. Breeder mice were fed a diet containing Prostandim (calculated dose of 457 mg/m^2^ which is equivalent to 675 mg/day for a 60 kg adult human) and the diet was continued after birth for six weeks. A second part of the study assessed the diet over six months. There was a significant reduction in thiobarbituric acid reactive substances (TBARS, a measure of oxidative stress and lipid peroxidation); however, there was no reduction in serum CK or histological disease parameters. There was also no change in the *gastrocnemius* muscle when assessed by magnetic resonance imaging. The lack of significant changes in pathology translated into a lack of functional improvement with voluntary exercise. While Chinese herbs may contain some glucocorticoid activity, these studies suggest that this activity is not enough to reduce dystrophic pathology or improve muscle function.

Chinese herbal supplements are not currently regulated by the FDA or the Australian Therapeutic Goods Administration (TGA). Many of the imported supplements could be contaminated with pesticides that are not legal in Western countries [106,107], or be contaminated with heavy metals such as lead, mercury, cadmium and thallium [108,109]. Regular users of Chinese herbal medicines may have an increased risk of developing cancers/diseases of the kidneys and other organs of the urinary tract [110,111] and heavy metal poisoning [107,108,109,112,113]. Many reports suggest that Western countries such as the USA, Australia and the United Kingdom should improve quality standards and policies regarding the sale of supplements, especially those from non-Western countries. Practitioners of Chinese herbal medicine should be well versed in pharmacology and potential side effects [114,115,116].

Another major consideration around using Chinese herbal medicine to treat DMD is their glucocorticoid activity. While studies indicate that levels were not enough to translate into clinical improvements [103,104], most DMD patients are prescribed corticosteroids and the Chinese herbs could interfere with this regime or cause adverse cumulative effects.

Any positive findings on the use of Traditional Chinese Medicine to treat DMD are currently speculative, and there may be risks associated with their use. It is therefore recommended that DMD patients do not supplement with Chinese herbs.

### 3.4. Green Tea Extract

Green Tea and Green Tea Extract (GTE) contain high levels of polyphenols which are predominantly comprised of catechins including gallocatechin (GC), epigallocatchin (ECG), epicatechin (EC) and epigallocatechin gallate (EGCG) [117,118], with EGCG being the most abundant and accounting for most of the medicinal properties [119,120]. Green tea has been extensively studied over the past couple of decades due to its reported medicinal properties, including its antioxidant [120,121] and anti-inflammatory properties [120,121]. These antioxidant and anti-inflammatory effects are in part mediated by reduced NF-κB pathway signaling [122,123]. The NF-κB pathway has critical roles in inflammation, immunity, cell proliferation, differentiation, and survival. NF-κB and its downstream pro-inflammatory cytokine targets are up-regulated in muscles of DMD patients and in *mdx* mice [124,125,126]. GTE has cardioprotective properties [127,128,129] which could be beneficial in DMD patients as cardiomyopathy is the leading cause of mortality in DMD.

Five studies have assessed the efficacy of GTE in *mdx* mice. The first assessed the efficacy of treating pregnant and newborn mice with 0.01% and 0.05% GTE in the feed for four weeks [130]. Overall, the treatments had no effect on the bodyweight of the mice. Both dosages reduced necrosis in the *Extensor digitorum longus* (*EDL*) muscle (by approximately 15%) but there was no change in the *soleus* muscle. Other muscles such as the larger hind limb muscles (*quadriceps, gastrocnemius* and *tibialis anterior*) were not assessed. This study was a brief, proof-of-principle study and no other measurements of pathology or muscle function were collected.

A second study compared GTE (0.05 and 0.25% wt/wt in the feed) or ECGC (0.1% wt/wt in the feed) in *mdx^5Cv^* mice [131]. The high dose GTE diet significantly reduced muscle necrosis in the *EDL* muscle (by approximately 10%) after five weeks of treatment; however, neither diet improved *soleus* muscle pathology, which is consistent with the previous study [130]. Multiple measures of muscle function were evaluated and improvements were seen for all substances tested, with the ECGC group performing slightly better than the GTE groups in terms of force-frequency relationships (approximately 10% improvement), fatigue resistance (22% improvement) and twitch tensions (33%–50% improvement) [131]. This study cemented the findings in the original study [130] and expanded them by including physiological measurements of muscle function. Fibrosis, cardioprotection or other measures of pathology (such as serum CK activity) were not evaluated and the larger hind limb muscles (*tibialis anterior, gastrocnemius* and *quadriceps*) were not assessed.

A more physiological approach was taken in a study that fed three-week-old *mdx* mice 0.5% GTE for three weeks while allowing voluntary wheel running exercise [132]. The premise behind the voluntary exercise was that exercise increases antioxidant capabilities in non-disease states; however, exercise in DMD exacerbates the phenotype [133,134,135]. This study showed that *mdx* mice fed GTE ran 94% further each day than untreated *mdx* mice. Hypertrophy was observed in the *gastrocnemius* muscle and the heart. While a gain of muscle mass can be beneficial in the hind limbs, hypertrophy in an already compromised DMD heart can be detrimental and therefore cardiac studies will need to be performed in the future. In the absence of voluntary exercise, serum CK was reduced by approximately 50% in the GTE treated mice indicating decreased muscle fiber damage. Histology data were not presented, although the authors indicate that they are consistent with previous studies.

In a further extension to the published work, *mdx* breeder mice and pups were fed GTE (0.25% and 0.5% in the feed) for 42 days [136]. Serum CK levels were significantly reduced after 42 days by approximately 80% by both GTE diets when compared to the control diet; however, no change was observed after 28 days. Histology showed that the GTE decreased the area of regenerating muscle fibers by around 15%, and this was accompanied by an approximately 10% increase in the number of normal/non-damaged fibers in the *TA* muscle. This finding is significant as the other studies focused on the smaller muscle groups, including the *EDL* and *soleus*. There were no changes in total immune cell infiltration and macrophage infiltration in the *TA* at either 28 or 42 days, which is surprising as GTE is considered a potent anti-inflammatory compound.

The most recent study administered 5 or 10 mg/kg GTE to *mdx* mice at three weeks of age via daily subcutaneous injections for a period of five weeks [137]. Mice treated with the low dose (5 mg/kg) showed a 50% reduction in serum CK; however, mice treated with the higher dose showed no significant improvement. This improvement in CK in the 5 mg/kg group was translated to a 30% improvement in locomotor activity. Surprisingly, the greatest functional improvement was observed in the high dose group despite there being no reduced pathology. The subcutaneous GTE injections did not reduce muscle fibrosis. This was not surprising as the experiment was completed when the mice were only eight weeks old, and fibrosis is a measure of chronic, long-term damage.

There is still much to learn about GTE and the potential benefits it has in DMD. The pre-clinical data suggest that it could aid in preventing the early stages of necrosis in DMD. Extended treatment schedules should be evaluated to determine if there are long term benefits. There is a high degree of variability in these studies, from the types of GTE used, how the GTE was purified, the dosages used and muscles assessed.

A registered clinical trial is currently recruiting DMD patients to assess GTE therapy in a double-blind, placebo-controlled, randomized study. Results are not expected until 2017 [138]. A second clinical trial using GTE to treat the milder Becker Muscular Dystrophy is also in the recruitment phase [139]. It is important to note that there have been safety concerns regarding green tea extracts causing liver damage/hepatotoxicity [140]; however, a more recent meta-analysis concluded the potential for these effects is extremely low [141]. Overall, the efficacy of GTE for treating DMD will remain unknown until completion of the aforementioned clinical trials.

## 4. Targeting Inflammation

Muscle inflammation is a coordinated multi-step process which is highly regulated and involves many cell types and myogenic factors. Within two hours of muscle injury, neutrophils invade the muscle reaching peak concentrations between 6 and 24 h post-injury and then declining rapidly [142]. The next cell type to invade are phagocytic M1 macrophages which reach peak levels from 24 h to two days post-injury then decline in number [143,144]. Both neutrophils and M1 macrophages release pro-inflammatory cytokines, including tumor necrosis factor alpha (TNF-α), interleukin 1 beta (IL-1β) and interleukin 6 (IL-6), which promote cell lysis and phagocytosis of necrotic myofibers, clearing the way for muscle regeneration. Two days post-injury neutrophils and M1 macrophages are replaced with a second wave of macrophages, M2 macrophages, which release anti-inflammatory cytokines such as interleukin 10 (IL-10) and transforming growth factor beta (TGF-β) to attenuate the immune response and promote tissue repair [145]. These M2 macrophages are comprised of three subtypes, M2a macrophages which promote wound healing and tissue repair, M2b macrophages which release anti-inflammatory cytokines to halt the immune response and M2c macrophages which release cytokines to inactivate the M1 macrophages and promote cell proliferation (reviewed in [142]). These M2 macrophages reach peak concentrations at approximately four days post-injury, but persist for many days until regeneration is complete.

Acute inflammation is an essential process required for muscle repair and regeneration following muscle injury. By contrast, chronic inflammation is vastly different and is thought to be detrimental in DMD [58,83,144,146,147]. In DMD patients and *mdx* mice the onset of muscle histopathology coincides with the onset of inflammation [126,148,149], suggesting inflammation plays a role in the disease pathology. During the early stages of *mdx* pathology, inflammation is similar to that observed in acute muscle injury, with an initial influx of neutrophils and M1 macrophages to clear the damaged tissue [150]. However, unlike acute muscle injury, the influx of M1 macrophages is accompanied by an influx of M2a macrophages which are usually only present during the later stages of inflammation [144,145,151]. In addition, these M2a macrophages, which typically promote wound healing and tissue repair, serve a different function in *mdx* mice by inhibiting nitric oxide mediated cell lysis from M1 macrophages and promoting fibrosis through the production of arginase. These M2 macrophages may also activate T-cells which contribute to muscle damage through perforin mediated cell lysis. Overall in *mdx* mice, the inflammatory response is perturbed from what is normally observed in acute muscle injury and more closely resembles other neuromuscular disorders such as inflammatory myopathies, where inflammation plays a key role in disease pathology [60,152]. As inflammation has a central role in exacerbating dystrophic pathology, compounds with anti-inflammatory properties are attractive therapeutic agents.

### 4.1. Soybeans

Like most legumes and plants, soybeans contain active biological compounds with anti-inflammatory and other biological functions that can potentially treat disease. Genistein is a soy isoflavone which is responsible for biological functions including inhibiting ROS [153], and pro-inflammatory mediators such as NF-κB, TNF-α, STAT-1, MAPKs and other cytokines [154,155,156,157,158,159]. The safety profile of genistein is positive, with low oral toxicity and it is available in the United States as a natural supplement [160].

Genistein treated *mdx* mice (daily intraperitoneal injection with 2 mg/kg) showed a 25% increase in forelimb strength [161]. This increase in strength translated to an approximately 40% decrease in necrosis in the *biceps* muscle and around a 50% increase in the area of regenerating fibers. This correlated with an increase in the number of myogenin positive nuclei, although there was no significant change in the amount of developmental myosin heavy chain staining. Serum CK levels were reduced by around 20% in the genistein treated mice, again indicating reduced muscle pathology. Genistein treated *mdx* mice showed significantly reduced NF-κB DNA binding activity, suggesting a possible inhibitory effect. Expression of pro-inflammatory molecules TNFα and phospho-JNK (related to the MAPK signaling pathway) were also significantly reduced. The improved function and reduced necrosis and serum CK in the genistein treated group were comparable to those in *mdx* mice treated with methylprednisolone (0.75 mg/kg/day intraperitoneally), one of the corticosteroids used to treat DMD. This is a positive finding and suggests genistein could reduce pathology and improve muscle function in DMD. Studies in DMD patients are required to determine if genistein can provide the same therapeutic effect as prednisolone without the adverse side effects; however, to date no clinical trials of genistein for DMD have been registered.

Another study evaluated the use of Bowman-Birk Inhibitor (BBIC), a serine protease inhibitor derived from soy, in *mdx* mice [162]. BBIC has anti-inflammatory properties and inhibits the release of neutrophil and mast cell proteases including neutrophil elastase and mast cell chymase [163,164,165,166]. These enzymes are produced by immune cells, are involved in TGF-β activation and fibrosis, and are implicated in progression of DMD pathology [167,168,169]. Previous studies have shown beneficial effects in *mdx* mice from inhibiting serine proteases [170,171,172,173]. Therefore, this study aimed to investigate whether BBIC, a naturally occurring compound with high systemic bioavailability [174,175] and low toxicity [175,176,177], could ameliorate DMD pathology. BBIC (1% in mouse chow for 12 weeks) reduced a variety of pathological measures. Fibrosis was reduced by approximately 12%, and the percentage of damaged myofibers (visualised by Evans blue dye) was reduced from 8.3% (control) to 3.1%. Serum CK was reduced approximately three fold in the BBIC treated group indicating reduced pathology [162]. The improvement in pathology was accompanied by improved muscle function. Absolute tetanic force in the *EDL* was increased; however, when normalized for *EDL* size, there was no change in specific force. Recovery time from eccentric contraction-induced injury was decreased 25.7% by BBIC treatment. These results show BBIC treatment reduces pathology and improves muscle function in *mdx* mice.

Together, these studies show that components derived from soybeans have potential to modulate the immune response and chronic inflammation in DMD. Whilst the results in the *mdx* mice are promising, both trials tested a single dosage and evaluated outcomes at a single time-point and further studies are needed to determine their optimal dosage. No human clinical trials have been reported using soy nutraceuticals in DMD patients and none are recruiting or registered on the Clinical Trials (clinicaltrials.gov) website.

### 4.2. Curcumin

Curcumin (1-7 bis(4-hydroxy-3-methoxyphenyl)-1,6-heptadiene-3,5-dione) [178] is a compound extracted from the roots of the turmeric plant which displays anti-inflammatory [179,180,181,182] and antioxidant properties [183,184,185,186]. Curcumin significantly reduces NF-κB activation by preventing translocation of the p65 subunit of NF-κB to the nucleus [187,188,189]. NF-κB is a pro-inflammatory mediator and is involved in the production of NO in oxidative stress and therefore is a key contributor to DMD pathogenesis.

Two studies have assessed the use of curcumin in *mdx* mice. The first placed *mdx* mice on a diet containing 1% (*w/w*) curcumin compared to a control, standard chow diet [190]. They found that NF-κB activity was unchanged in the *soleus* and *diaphragm* muscles from curcumin fed mice, indicating that 1% dietary curcumin was not sufficient to inhibit NF-κB activity in dystrophic muscle. Interestingly, curcumin treated *mdx* mice showed improved muscle contractile properties compared to controls. Although this study was an informative pilot, it did not address the effect of curcumin on disease pathology parameters in the *mdx* mice and only a single curcumin dosage was investigated [190].

The second study delivered 0.1 mg/kg, 0.5 mg/kg or 1 mg/kg intraperitoneally to *mdx* mice daily for 10 days starting at 18 days of age and assessed if muscle pathology was improved [191]. Curcumin reduced muscle damage in *mdx* mice as evidenced by a decrease in Evans blue dye staining, reduced skeletal muscle necrosis, reduced variation in muscle fiber size, and fewer central nuclei. These results were presented as representative images and no quantification was performed, so it is not clear if the improvements are present across the total muscle cross section. The highest dose of curcumin (1 mg/kg) significantly improved parameters of muscle function including grip-strength (by approximately 10%) and ability to hang when suspended on wire (by approximately 50%). The inflammatory markers TNF-α and Il1β were reduced in the serum of curcumin-treated mice, suggesting that the reduced pathology and improved muscle function is via an anti-inflammatory mechanism. In contrast to the earlier report in which NF-κB activity was not reduced [190] this study showed NF-κB was inhibited by curcumin in a dose-dependent manner. This could be due to higher bioavailability of intraperitoneally delivered curcumin compared with oral administration and this would need to be addressed if trialed in DMD patients [192,193,194].

Curcumin has not yet been tested in clinical trials for DMD or other neuromuscular disorders; however, it is available as a supplement due to its anti-cancer properties (reviewed in [195]). The main impediment to using curcumin to treat disease is that it has very low oral bioavailability [196]. Curcumin is rapidly metabolized in the liver and intestine and high concentrations of curcumin cannot be maintained in the plasma or tissues such as skeletal muscle. Aerosols [192] and nanoparticles [193,194] have been used to enhance bioavailability but this technology has not yet been trialed in *mdx* mice or DMD patients. Curcumin toxicity studies have not been reported; however, dosages of 0.9 to 3.6 grams per day for four months (not enough to increase levels in muscle and plasma) resulted in nausea and diarrhea [197]. Based on these data, curcumin has anti-inflammatory properties that could be of some benefit in treating DMD but further studies are required to evaluate toxicity, dose and delivery methods.

### 4.3. Resveratrol

Resveratrol (3,5,4-trihydroxy-*trans-*stilbene), a polyphenol found in grapes has been labelled the “fountain of youth” for its ability to increase lifespan in yeast, worms, flies and mice [198,199,200,201]. Many pathways are impacted by resveratrol and include those involved in carcinogen metabolism, cellular proliferation, inflammation, cell cycle regulation and apoptosis [202,203,204,205,206,207]. Many of these actions result from the activation of sirtuin 1 (Sirt1) by resveratrol. Sirt1 is the most well characterized member of the sirtuins, an evolutionary conserved family of NAD+ dependent (class III) histone and protein deacetylases. Resveratrol activates Sirt1 by allosterically binding to the N terminus [208]. Sirt1 activation results in the deacetylation of an array of signaling targets which regulate physiological processes in multiple tissue types (reviewed in [209]).

In C2C12 muscle myoblast cells, resveratrol increases myoblast differentiation by upregulating myogenic regulatory factors such as *MyoD* and *myogenin* [210], and increases glucose uptake and glucose transport through activation of AMP-activated protein kinase (AMPK) [211]. In addition, resveratrol treatment induced myoblast apoptosis [212] and enhanced cell survival [213,214].

These in vitro studies led to in vivo studies assessing the potential of resveratrol to treat muscle pathology in the *mdx* mouse. The reported studies treated with different amounts of resveratrol and assessed different outcome measures. Resveratrol treatment (4 g/kg of feed) of *mdx* mice for 32 weeks starting at nine weeks of age reduced *biceps femoris* muscle fibrosis by approximately 50% [215]. However, inflammatory cells were not reduced and mRNA for the inflammatory cytokines *TNF-α*, *IL-1β*, *TGF-β1*, and *TGF-β2* was upregulated in the *biceps femoris* muscle of *mdx* mice compared with that of control mice [215]. This finding was surprising because resveratrol has potent anti-inflammatory effects in other studies [216,217,218]. Markers of oxidative stress were reduced including NADPH oxidase but SOD1 was the only antioxidant that was significantly increased with resveratrol treatment [215]. It is possible that resveratrol was less effective than expected because the treatment only began when mice were nine weeks old, well after the acute onset of inflammation and pathology in the *mdx* mouse which occurs around three weeks of age. After this initial bout of damage, *mdx* disease pathology is reduced to chronic low levels of damage and inflammation by eight weeks of age [69]. As the period of peak inflammation was missed, this could explain why resveratrol treatment did not reduce inflammatory cells and cytokines. Functional parameters were not assessed in this study.

A second study fed one-month-old *mdx* mice either 100 mg/kg/day or 400 mg/kg/day resveratrol for eight weeks [219]. In the 100 mg resveratrol group there was a significant reduction in bodyweight and the weights of *EDL*, *soleus* and *TA* muscles [219]. This study focused on assessing muscle function and found an approximately 20% increase in fatigue resistance in the *soleus* muscle from mice in the 100 mg/kg/day resveratrol group [219]. The 100 mg/kg/day resveratrol treatment did not protect the *EDL* or *soleus* muscles from contraction-induced injury [219]. There was a high mortality rate in the 400 mg/kg/day resveratrol treated group which could indicate that resveratrol is toxic at this dosage [219]. Heart weights were increased in the surviving animals and is of concern given progressive cardiomyopathy is the leading cause of death in DMD patients [14]. Future studies will need to assess the effect of resveratrol on cardiac pathology in *mdx* mice to ensure that it would not worsen the phenotype already present.

Another study began treating *mdx* mice at five weeks of age, during the acute period of disease pathology, with 10, 100 or 500 mg/kg resveratrol delivered via daily oral gavage for 10 days [216]. Rather than looking for improvement in muscle pathology they screened to determine which dose produced the highest up-regulation of *Sirt1* gene expression. Sirt1 in turn activates Peroxisome proliferator activated receptor Gamma Coactivator 1 alpha (PGC-1α). PGC1α up-regulation reduces pathology and improves muscle function in *mdx* mice [219,220,221]. This study showed that the 100 mg/kg dosage was the only dosage that significantly up-regulated Sirt1 gene expression; however, this was not translated into an increase in protein expression. As resveratrol activates Sirt1 by allosterically binding to the N terminus it would have been more informative to measure Sirt1 activity, as increased Sirt1 activity does not necessarily result from increased Sirt1 gene expression [213]. The 100 mg/kg resveratrol treatment significantly decreased immune cell infiltration in the *gastrocnemius* muscle of *mdx* mice over the 10 days treatment period [216]. There was no reduction in gene expression of the pro-inflammatory cytokine *TNF-α*. This was a preliminary pilot study, so other parameters of pathology or muscle function were not analyzed.

Although resveratrol has beneficial effects on muscle pathology and function in *mdx* mice, the studies do not contain detailed analyses of muscle pathology, muscle function and molecular signaling pathways. The dosages and dosage regimes differ among the studies, as do the analyses performed. There are some hurdles to overcome before resveratrol can be used to treat DMD patients. Although resveratrol solubility is excellent (at least 70% absorption of a 25 mg dose in healthy patients) [222], the bioavailability is very low and resveratrol was only detected in trace amounts in patient plasma after a 25 mg dose. Levels were high in the urine suggesting that rapid metabolism by the liver and or intestine limits the bioavailability of resveratrol [222]. The safety profile of resveratrol is excellent, with a trial in healthy volunteers finding no adverse side effects with a single 500 mg dose of resveratrol [223]. Another study found no adverse side effects with a high dose of resveratrol (1000 mg/day) in overweight, older adults for a 90 days period [224]. A number of groups are exploring ways to enhance the bioavailability of resveratrol [225,226,227,228]. More pre-clinical studies are required to determine if resveratrol could be a useful DMD therapy.

### 4.4. Increasing Nitric Oxide Availability

The loss of dystrophin results in the consequent loss of a number of proteins which are normally anchored to the sarcolemma through their interaction with dystrophin. One such protein is neuronal nitric oxide synthase (nNOS). In the *mdx* mouse and DMD patients the loss of sarcolemmal nNOS results in a susceptibility to ischemia especially following mild exercise such as day-to-day limb movement. Additionally, localization of nNOS to the sarcolemma has been implicated in protection from the damaging effects of inflammation and oxidative stress, fat deposition, fibrosis and activation of satellite cells [73,229,230,231,232]. Under normal circumstances muscle derived nitric oxide (NO) attenuates the α-adrenergic vasoconstriction in exercised skeletal muscle which acts to optimize blood flow; this is referred to as functional sympatholysis [233,234,235]. This protective mechanism is defective in working skeletal muscle of *mdx* mice, Becker (BMD) and DMD patients resulting is ischemic damage [236,237,238]. In dystrophic muscle, repeated episodes of ischemia have been linked to muscle fatigue and increased damage that results from everyday muscle usage [72,239]. Other features of the dystrophic phenotype in skeletal muscle that may be related to altered blood flow include microvessel spasms which contribute to hypoperfusion injury and subsequent fatigue [240].

An extensive body of pre-clinical and clinical data show that increasing NO availability by targeting the NO-cyclic guanosine 3′,5′-monophosphate (cGMP) pathway is beneficial in dystrophic skeletal muscle. Pharmacological approaches to correct NO availability in dystrophic muscle include agents such as NSAIDS and phosphodiesterase 5 (PDE5) inhibitors which improve blood flow and slow disease progression resulting in reduced necrosis and inflammation [230,241,242]. PDE5 inhibitors, such as tadalafil and sildenafil, prolong the half-life of (cGMP) which is a downstream target of NO. In *mdx* mice and DMD patients these drugs alleviate the ischemia that is associated with functional sympatholysis and normalize exercise induced increases in skeletal muscle blood flow [240,241]. Corticosteroids such as prednisone or deflazacort that are in routine clinical use do not offer any protection from functional ischemia [241]. This is also true of angiotensin-converting enzyme inhibitors (Lisinopril) or angiotensin receptor blockers (losartan) which are used to delay heart failure [241]. This all points to a major unmet clinical need to address the deleterious effects associated with decreased blood flow. In this context, two nutraceutical compounds aimed at increasing available NO include sodium nitrate, contained within beetroot juice, and l-arginine.

#### 4.1.1. Beetroot Juice

Beetroot juice is a source of sodium nitrate which shows activity as an inorganic NO donor. In the only study of its kind to date Nelson et al. [238] hypothesized that orally delivered sodium nitrate, in enriched beetroot juice, would rescue defective sympatholysis in BMD patients. This study considered two different treatment protocols. Firstly, they conducted a single dose open label trial in 11 BMD adults where patients received 8.4 mmol inorganic nitrate in 140 mL of beetroot juice with a handgrip exercise protocol conducted 3 h after administration. The second trial was more rigorous in design being conducted as placebo (beetroot juice depleted of sodium nitrate) cross over trial with a two-week washout conducted in a subset of six patients with a similar dose and exercise protocol. This study demonstrated that a single dose of inorganic nitrate alleviates functional ischemia in BMD muscle by restoring sympatholysis in 9 out of 11 patients after 3 h.

While the study by Nelson et al. [238] was not designed to test the effect of inorganic nitrate on the progression of the disease it provides an excellent basis for further considering inorganic nitrate therapy. The use of inorganic nitrate has a key advantage over organic nitrates as there are no reports of the development of tolerance. A number of organic NO donors like glyceryl nitrate have been used clinically but they have been limited in their use, especially in chronic treatment regimes, by the development of tolerance [243,244]. This unlikely to be a feature of inorganic nitrate administration but needs further experimental confirmation. It is unclear based on this single dose acute study whether chronic administration will sustain improved blood flow regulation but sodium nitrate should be considered for future trials given the promising data to date [238]. For all agents that have been shown to restore normal blood flow following activity, an important consideration is whether this translates to preserved dystrophic skeletal muscle and slowing of disease progression.

#### 4.1.2. l-arginine

Several pre-clinical studies have assessed the use of l-arginine in *mdx* mice. The first study used two different delivery methods to administer the l-arginine to *mdx* mice; daily intraperitoneal injections (400 mg/kg bodyweight/day) for 28 days and osmotic pumps with doses of 200 and 400 mg/kg body weight/day of l-arginine for four and eight weeks [245]. Each treatment was compared to a saline control. Central nucleation was unchanged with the four-week l-arginine treatment; however, Evans Blue Dye positive myofibers were reduced in the *quadriceps.* l-arginine treatment did not significantly alter the functional parameters isometric twitch, maximum tetanic force or specific force in *quadriceps*. Interestingly, the *EDL* muscle from the l-arginine treated *mdx* mice was protected from contraction-induced injury as the decrease in force was significantly less in *EDL*s from l-arginine–treated *mdx* mice than in *EDL*s from *mdx* mice receiving saline treatment. Total utrophin protein was increased approximately 21% by l-arginine and there was increased utrophin at the sarcolemma, indicating the therapeutic effects could be due to compensation from utrophin.

A second study compared the effects of l-arginine in conjunction with the corticosteroid deflazacort, with deflazacort alone and a placebo in four-week-old *mdx* mice [246]. Deflazacort was administered subcutaneously (1.2 mg/kg/day in methylcellulose) and l-arginine was administered in the drinking water (at a concentration of 0.375% *w/v*). Mice were treated for 21 days and the end points were as follows: protocol 1: acute, after 24 h of voluntary exercise; protocol 2: short-term, 24 h of voluntary exercise followed by four days recovery; and protocol 3: long term, treatment regime followed by three months without treatment. In the short term protocol the proportion of Evans Blue Dye positive myofibers in the *quadriceps* was lowest in the deflazacort plus l-arginine group. *Myf5* gene expression was used as a measure of regeneration. *Myf5* was significantly reduced by deflazacort treatment, and was further reduced in the deflazacort plus l-arginine group. Finally in the long-term protocol the mice treated with deflazacort ran ~2.5 times further than the placebo group, and the group treated with deflazacort plus l-arginine ran further than those treated with deflazacort alone. This study only used Evans Blue Dye to measure damage and did not measure necrosis or fibrosis.

Another study administered l-arginine (200 mg/kg bodyweight) intraperitoneally to five-week-old *mdx* mice for two weeks [247]. Histological analysis of the *diaphragm* revealed a 20% decrease in necrosis and an increase in regenerating myofibers, evidenced by central nucleation. Inflammatory cytokines TNFα, IL-1β, and IL-6 were reduced in the l-arginine treated *mdx diaphragm* when compared to the saline treated *mdx* group. Utrophin protein was increased approximately 1.5–2 fold with l-arginine treatment; however, expression of nNos was unchanged. This study only assessed the *diaphragm;* none of the hind limb muscles were examined for changes in pathology.

A final pre-clinical study assessed the effects of arginine butyrate (250 mg/kg/day administered intraperitoneally) alone and in conjunction with prednisone (1 mg/kg/day slow-release subcutaneous pellets) in three-month-old *mdx* mice for six months [248]. Treatments were compared to saline control and to prednisone alone. Forelimb grip strength was significantly improved in the arginine butyrate treated *mdx* mice and in the combined treatment group (prednisone plus arginine butyrate) when compared to the saline treated controls. Fibrosis was significantly reduced in the *gastrocnemius* of the arginine butyrate treated *mdx* mice and was unchanged in both the prednisone and the arginine butyrate plus prednisone treatment. Serum creatine kinase was similar in the three groups indicating that pathology is not significantly reduced with arginine butyrate treatment. In addition, there were no significant differences in cardiac histology or cardiac function with the arginine butyrate or combined treatments.

Overall these pre-clinical studies demonstrate that l-arginine could have some benefit in DMD; and as such an open label, single center, proof-of-concept clinical trial was performed to see if l-arginine provided DMD patients with any therapeutic benefits. Five ambulant DMD patients between the ages of 7 and 10 years were treated with a combination of l-arginine (3 × 2.5 g/day) and metformin (2 × 250 mg/day) for 16 weeks [249]. Mitochondrial electron transport chain proteins were elevated in the treatment group and oxidative stress was reduced. Motor function measures and the mean 2 min walking distance improved in four of the five treated patients; however, the oldest and most severely affected patient continued to decline despite the treatment.

The use of NO donors to restore NO signaling in dystrophic muscle is attractive as they correct deficiencies in vascular regulation [242] and can also act to inhibit histone deacetylases which improves differentiation and regeneration [250]. This should be balanced by the potential for hyper-nitrosylation of ryanodine receptors that can reduce muscle force generation capacity [251,252]. Importantly, none of these therapeutic options correct the localization of nNOS to the sarcolemma but rather appear to enhance residual cytosolic nNOS activity. Nonetheless interventions that increase muscle derived NO have therapeutic potential.

### 4.5. Taurine

Recent studies demonstrate that cysteine precursor antioxidants such as N-acetyl cysteine (NAC) and l-2-oxothiazolidine-4-carboxylate (OTC) reduce dystropathology in the *mdx* mouse model of DMD [253,254]. This therapeutic benefit likely occurs through increased synthesis of the amino acid taurine. To test if direct supplementation with taurine would be beneficial in *mdx* mice, 18-day-old mice were treated with 2% taurine in the drinking water for 24 days [220]. This taurine supplementation regime resulted in a 1.2 fold increase in taurine content in the *mdx* muscle and a four-fold increase in the *mdx* liver. Taurine supplementation in *mdx* mice resulted in significantly improved grip strength, restoring function to wildtype levels, and significantly restored parameters of ex vivo muscle function in the *EDL* muscle including specific force, time to peak force and half relaxation time. The peak twitch force and rate of maximal force production were not significantly altered. Overall these results are highly promising and encourage future studies into the effects of taurine supplementation for treating DMD. The safety profile of taurine supplementation is quite limited; however, one study has shown a serious adverse effect when treated with high doses (200 mg/kg/day) for up to 50 months. One patient required hospitalization for hypersomnia, and other patients had milder side effects included moderate fatigue, somnolence, cognitive change and mild insomnia [255]. As these side effects occurred at high doses more research is required before supplementation can be recommended.

### 4.6. Vitamin D

Vitamin D is a key component for bone health and is obtained from the diet (mainly present in fatty fish or vitamin D enriched foods such as dairy, soy milk and orange juice) or can be synthesized in the body by converting 7-dehydrocholesterol present in the skin following ultraviolet (UV) light exposure. Vitamin D promotes calcium absorption from the small intestine. The active vitamin D metabolite, 1,25D binds to the vitamin D receptor in intestinal cells and stimulates formation of calbindin which binds calcium and influences the calcium channels [256]. Long-term corticosteroid use is detrimental to bone health as they increase urinary calcium loss and interfere with vitamin D metabolism [257]. As approximately 78% of DMD patients are vitamin D deficient [258], patients are advised to supplement with both calcium and vitamin D to maintain bone health and prevent fracture [3]. Data from Duchenne Connect, an online patient registry where patients self-report data, revealed that vitamin D supplementation significantly increased the probability of walking at age 12 [259]. In addition, a second study evaluated the efficacy of calcifediol (25-OH vitamin D3) and adjustment of calcium intake in 33 DMD patients (aged between 5 and 15 years) who had undergone at least six months of corticosteroid treatment [260]. Patients were observed for one year and then underwent two years of calcifediol treatment (0.8 mcg/kg/day) (plus adjustment of the dietary calcium intake to the internationally recommended daily allowance). During the year of observation, calcium levels were lower than normal; however, these values were increased to normal levels at the end of the calcifediol treatment. Similarly, 25-OH D levels were significantly increased with the treatment. At the baseline and during the year of observation, markers of bone resorption including urinary collagen type 1 cross-linked N-telopeptide (NTx) and collagen type 1 cross-linked C-telopeptide (CTx) were higher than normal; however, both were significantly decreased with the calcifediol treatment. Bone mineral content (BMC) and bone mineral density (BMD) scores decreased during the observation period; however, after the treatment 22 patients had significant increases in BMC. Overall these results are highly encouraging for patients and indicate they should supplement with vitamin D and calcium to maintain bone health and delay loss of ambulation.

Finally, a retroactive study over a 16-year period from 1998 till 2014 assessed the efficacy of vitamin D supplementation in DMD [261]. They found that despite recommendations for DMD patients to supplement with vitamin D there is still a high prevalence of vitamin D deficiency/insufficiency in DMD patients. In addition, they tested the following maintenance doses of vitamin D: daily (200, 400, 800, 1000 or 1500 IU) or weekly (3000 or 6000 IU). The main findings were that the 1500 IU dose was required to achieve optimum serum 25(OH)D vitamin D levels. Moreover, a replenishment regimen of 6000 IU daily for three months achieved optimal vitamin D levels in 84% of the patients compared to only 52% on 3000 IU per day. The main conclusion of this study was that patients require monitoring every six months to maintain optimum serum vitamin D levels. Overall these results are highly encouraging for patients to supplement with Vitamin D to maintain bone health and prevent from fracture-associated loss of ambulation.

## 5. Discussion

Complementary and alternative medicine is becoming increasingly popular in Western countries such as the United States and Australia and is a multi-billion dollar per year industry [53]. This review summarized the available data on nutraceuticals which have been trialed clinically in DMD patients or in the pre-clinical *mdx* mouse model. Although some nutraceuticals such as co-enzyme Q10, components of soybeans and taurine show promise, others such as Chinese herbal medicine have limited or no beneficial effects (Table 1).

The main challenge when comparing studies that have used nutraceutical interventions in the *mdx* mouse is the lack of consistency. Studies use different dosages, treat mice at different ages, for different periods of time, assess different muscles or only assess pathology or function independently of each other (Table 2). This highlights the need to follow pre-clinical standard operating procedures when assessing any intervention be it nutraceutical or pharmaceutical. In addition, pre-clinical trials would benefit from directly comparing improvements in muscle function and performance with those achieved with corticosteroid treatment, to determine overall efficacy and translational potential. The Treat-NMD Neuromuscular Network has freely available standard operating procedures which have been developed by experts in the field to enable researchers worldwide to effectively compare results obtained from intervention studies and assess how their therapy compares to the standard corticosteroid therapy [263]. These standard operating procedures will also be beneficial when determining which compounds have scientific merit and should progress to human clinical trials.

It is important to note that the supplement industry is not regulated by the FDA (USA) or TGA (Australia) and therefore supplement manufacturers do not have to meet the stringent requirements that the pharmaceutical industry has to conform to. Many studies have highlighted the need for nutraceutical supplements, especially those obtained from non-Western countries, to meet safety and dosage standards [106,114,116]. As with any pharmaceutical, nutraceuticals have the potential to interact with current medications, so any supplementation in DMD patients should be cleared by the primary physician.

## 6. Conclusions

While nutraceuticals will never cure DMD they could have significant potential as complimentary therapies in counter-acting the damaging effects of chronic inflammation or oxidative stress. A number of nutraceuticals have clinical merit and more research into their therapeutic potential to treat DMD is justified. Whilst corticosteroids are the current therapeutic standard for treating DMD and can prolong ambulation and muscle function [20,21,25], many patients experience serious adverse side effects (see Introduction). If a nutraceutical could produce similar therapeutic benefits to corticosteroids without adverse side effects, it could provide many DMD patients with an improved quality of life as well as reduce costs associated with recurrent hospital visits to monitor and treat corticosteroid-induced side effects. For this to occur, research needs to focus on performing good quality peer-reviewed research using compounds that have scientific merit and thus separate the credible from the anecdotal conjecture.

## Figures and Tables

**Table 1:** Summary of findings from nutraceutical pre-clinical and clinical trials.

Nutraceutical	Mechanism	*mdx* Mouse	Human Trial
Co-enzyme Q10	Antioxidant	-	Initial study anecdotal [76]. Small-scale CINRG trial showed improvement in muscle function and promise in improving cardiac function [77]. Large scale CINRG trial ongoing [79].
Melatonin	Antioxidant	Reduced serum CK and improved muscle contraction times in *biceps* [89].	Reduced oxidative stress and serum CK [90].
Chinese herbal medicine	Antioxidant	-	Clinical trial reported only anecdotal evidence [92].
Prostandim (contains Chinese herbs)	Antioxidant	No improvement in serum CK, histology, MRI or muscle functional parameters [94].	-
Green Tea Extract	Antioxidant	Reduced necrosis in *EDL* but not *soleus* [119]. Some improvement in *EDL* muscle function [120,121]. Slight (10%) improvement in *TA* muscle [125]. Variable reduction in CK depending on administration route [126]. Some hypertrophy observed in heart	A trial is in recruitment phase, results expected in 2017 [221,262].
Taurine	Antioxidant	Improved fore limb grip strength, improved isometric force, reduced stretch induced damage, reduced protein thiol oxidation [220].	-
Soybeans	Anti-inflammatory	Genestein reduced serum CK, necrotic area and improved *biceps* muscle strength. Comparable to methylprednisolone [161]. Bowman Birk Inhibitor (BBIC) reduced fibrosis, serum CK and necrosis. Improved *EDL* muscle function [151].	-
Curcumin	Anti-inflammatory	Improved contractile properties [179]. Reduced necrosis, serum CK and central nuclei (not quantitated). Improved grip strength and hang time [180].	-
Resveratrol	Anti-inflammatory	Decreased fibrosis [204]. Reduced bodyweight and *EDL*, *soleus* and *TA* weights. Increased fatigue resistance in *soleus* [208]. Decreased immune cell infiltration in *gastrocnemius* [205].	-
Beetroot Juice	Increasing NO	-	Improved blood flow. Corrected deficient sympatholysis [238].
l-arginine	Increasing NO	Reduced percentage of Evans blue dye positive myofibers, protected from contraction induced injury [245]. Reduced percentage of Evans blue dye positive myofibers, reduced expression of improved exercise performance [246]. Reduced necrosis in *diaphragm* [247]. Reduced inflammatory cytokine expression. Reduced fibrosis, improved grip strength no change in serum CK [248].	
Vitamin D	Unkown	-	Increased probability of walking through age 12 [259]. Calcifediol decreased markers of bone resorption, increased bone mineral content and bone mineral density [260]. Maintenance dose to obtain optimum serum vitamin D levels is 1500 IU daily along with a 3-month replenishment dose of 6000 IU daily for 6 months.

Abbreviations: *Tibialis anterior (TA)*, *Extensor digitorum longus (EDL)*, Creatine kinase (CK), The Cooperative International Neuromuscular Research Group (CINRG).

**Table 2:** Summary of nutraceutical treatment regime and outcome measures in *mdx* pre-clinical trials.

Nutraceutical	Dose and Delivery Method	Mouse Age and Treatment Duration	Muscles Assessed
Melatonin [89]	Daily intraperitoneal injection (30 mg/kg) or subcutaneous implants (18 and 54 mg/kg)	Begin at 2 weeks of age, treat for 2 weeks	*Triceps*
Chinese Herbs—Prostandim [94]	457 mg/m^2^ prostandim in chow	Fed to pregnant mice and continued feeding until offspring were 6 weeks and 6 months old	*Gastrocnemius*, *TA*, *rectus femoris* and *hamstring*
Green Tea Extract (GTE) [119]	0.01% or 0.05% GTE in chow	Fed to pregnant mice and neonates until 4 weeks old	*Soleus* and *EDL*
Green Tea Extract [120]	0.05% or 0.025% GTE in chow	Began when 3 weeks old, continued until 4 or 7 weeks of age	*Gastrocnemius*, *plantaris*, *soleus* and *EDL*
Green Tea Extract [121]	0.5% GTE in chow	Began when 3 weeks old, continued until 6 weeks of age	*EDL*
Green Tea Extract [125]	0.25% or 0.5% GTE in chow	Began when 3 weeks old, continued until 4 or 6 weeks of age	*TA*
Soybeans—Genistein [150]	Intraperitoneal injection 2 mg/kg genistein daily or 3 times a week	From 5 weeks of age until 10 weeks of age	*Biceps* and *EDL*
Soybeans—Bowman Birk Inhibitor (BBIC) [151]	0.1% BBIC in chow	From 4 weeks of age until 16 weeks of age	*TA*, *quadriceps*, *diaphragm* and *EDL*
Curcumin [179]	1% curcumin in chow	From 3 weeks of age until 5 weeks of age	*Diaphragm* and *soleus*
Curcumin [180]	Daily intraperitoneal injections; 0.1, 0.5 or 1 mg/kg	From 2.5 weeks until 4 weeks of age	*EDL*
Resveratrol [204]	0.04% resveratrol in chow	From 9 weeks of age until 41 weeks of age	*Biceps*
Resveratrol [208]	100 or 400 mg/kg/day resveratrol in chow	From 4 weeks of age until 12 weeks of age	*Soleus, EDL*, *TA* and *gastrocnemius*
Resveratrol [205]	10, 20, 100 or 500 mg/kg resveratrol in chow	From 5 weeks of age until 6.5 weeks of age	*Gastrocnemius* and *TA*
Taurine [220]	2% taurine in drinking water	From 18 days old until 42 days old	*TA* and *EDL*
l-arginine [245]	Daily intraperitoneal injection (400 mg/kg bodyweight/day) or subcutaneous osmotic pump (200 and 400 mg/kg bodyweight/day)	8 weeks of age then treat for 28 days for injection 4 and 8 weeks for osmotic pumps	*Quadriceps*, *EDL*
l-arginine [246]	In the drinking water (0.375% *w/v*)	4 weeks of age and treated for 17 days, 21 days and 21 days followed by 3 months of no treatment	*Quadriceps*
l-arginine [247]	Daily intraperitoneal injection (200 mg/kgbodyweight/day	5 weeks of age and then treated for 2 weeks	*Diaphragm*
l-arginine [248]	Daily intraperitoneal injection (250 mg/kg bodyweight/day)	3-month-old mice for a period of 6 months	*Gastrocnemius*

Abbreviations: Tibialis anterior (TA), Extensor digitorum longus (EDL), Green tea extract (GTE).
